# The Potential of Normobaric Oxygen Therapy to Enhance Erythropoiesis, Reduce Oxidative Stress, and Modulate Immune Function in Colorectal Cancer Patients Undergoing Chemotherapy: Study Protocol for a Prospective, Randomized, Double-Blind, Placebo-Controlled Trial (NBO-ONCO)

**DOI:** 10.3390/jcm14145057

**Published:** 2025-07-17

**Authors:** Jacek Polański, Beata Jankowska-Polańska, Robert Dymarek, Olga Zajączkowska, Sebastian Makuch, Beata Freier, Dorota Kamińska, Edyta Pawlak, Adam Busławski, Jerzy Zwoździak

**Affiliations:** 1 Department of Internal Medicine, Occupational Diseases, Hypertension and Clinical Oncology, Wroclaw Medical University, 50-556 Wroclaw, Poland; jacek.polanski@umw.edu.pl; 2 Center for Research and Innovation, 4th Military Clinical Hospital, 50-981 Wroclaw, Poland; bpolanska@4wsk.pl; 3 Faculty of Medicine, Wroclaw University of Science and Technology, 51-377 Wroclaw, Poland; dkaminska@4wsk.pl; 4 Faculty of Physiotherapy, Wroclaw Medical University, 50-368 Wroclaw, Poland; 5 Clinical Trials Support Centre, 4th Military Clinical Hospital, 50-981 Wroclaw, Poland; ozajaczkowska@4wsk.pl (O.Z.); sebastian.mk21@gmail.com (S.M.); 6 Department of Clinical and Experimental Pathology, Wroclaw Medical University, 50-368 Wroclaw, Poland; 7 Regional Digital Medicine Centre, 4th Military Clinical Hospital, 50-981 Wroclaw, Poland; bfreier@4wsk.pl; 8 Laboratory of Immunopathology, Department of Experimental Therapy, Hirszfeld Institute of Immunology and Experimental Therapy, Polish Academy of Sciences, 53-114 Wroclaw, Poland; edyta.pawlak@hirszfeld.pl; 9 Department of Management and Logistics, WSB Merito University in Opole, 45-372 Opole, Poland; adam.buslawski@opole.merito.pl; 10 Department of Physical Culture and Safety Sciences, Academy of Applied Sciences, 33-300 Nowy Sacz, Poland; zwozdziak@wp.pl

**Keywords:** colorectal cancer (CRC), normobaric oxygen therapy (NBO), chemotherapy-induced anemia, oxidative stress, immune modulation, erythropoiesis, supportive cancer care, adverse effects, stress, anxiety, depression, psychological well-being, quality of life

## Abstract

**Background/Objectives:** Colorectal cancer (CRC) patients undergoing chemotherapy often experience anemia, oxidative stress, and immune suppression, significantly impacting their quality of life and treatment outcomes. Normobaric oxygen (NBO) therapy, which delivers oxygen at atmospheric pressure with an elevated oxygen concentration, has shown the potential to enhance erythropoiesis, reduce oxidative stress, and modulate immune function. However, its efficacy in CRC patients remains underexplored. This study aims to evaluate the effects of NBO exposures on (1) supporting erythropoiesis by measuring erythropoietin (EPO) levels and hypoxia-inducible factor 1-alpha (HIF-1α), (2) reducing oxidative stress and improving stress and emotional well-being, and (3) modulating immune function by assessing cytokine profiles. Secondary objectives include assessing the impact of NBO on patient-reported outcome measures (PROMs) such as stress, anxiety, depression, and quality of life. **Methods:** This is a prospective, randomized, double-blind, placebo-controlled clinical trial. A total of 254 CRC patients undergoing chemotherapy will be randomized 1:1 to receive either active NBO therapy (n = 127, study group) or placebo NBO therapy (n = 127, control group). The intervention will consist of 10 NBO sessions over five weeks. Primary outcomes include biomarkers of erythropoiesis, oxidative stress, and immune response. Secondary outcomes assess quality of life and psychological well-being. Data will be collected at baseline, mid-intervention, post-intervention, and during two follow-up visits (3 and 6 months post-intervention). **Results:** The study hypothesizes that NBO therapy will improve erythropoiesis, reduce oxidative stress, and enhance immune function in CRC patients, leading to improved quality of life and clinical outcomes. **Conclusions:** Findings from this trial may establish NBO as a novel supportive therapy for CRC patients undergoing chemotherapy.

## Simple Summary

Colorectal cancer is a major global health challenge, and patients undergoing chemotherapy often experience anemia, immune dysfunction, and oxidative stress, which can negatively affect treatment outcomes and quality of life. A simple and non-invasive approach like normobaric oxygen therapy may help by boosting red blood cell production, reducing inflammation, and strengthening the immune system. This study aims to evaluate its effectiveness in patients with colorectal cancer receiving chemotherapy, assessing its impact on erythropoiesis, immune function, oxidative stress, and psychological well-being. By exploring this potential therapy, we hope to uncover a safer and more accessible way to support cancer patients during treatment with chemotherapy. If proven beneficial, normobaric oxygen therapy could be integrated into oncology treatment protocols, enhancing patient outcomes and supporting overall quality of life.

## 1. Introduction

Colorectal cancer (CRC) is the third most commonly diagnosed cancer worldwide and the second leading cause of cancer-related deaths, accounting for over 1.9 million new cases and 935,000 deaths in 2020 [1]. The incidence of CRC is highest in developed countries, largely due to dietary and lifestyle factors such as high consumption of processed foods, red meats, and alcohol and physical inactivity [2]. According to the Global Cancer Observatory, CRC cases are expected to rise sharply by 2035, particularly in low to middle-income countries adopting Westernized diets [3].

The increasing incidence in these regions underscores the impact of lifestyle changes and the urgent need for preventive strategies [4]. CRC screening programs have successfully reduced mortality rates in developed nations by enabling the early detection and removal of precancerous polyps [5]. For instance, widespread colonoscopy screening in the United States has led to a significant decline in CRC incidence and mortality [6]. However, limited access to screening in developing countries contributes to diagnoses at more advanced stages, resulting in poorer outcomes [7].

Economically, CRC imposes substantial healthcare costs. Advanced cases requiring treatments such as chemotherapy, targeted therapies, and surgical interventions like liver resections place a significant financial burden on healthcare systems [8]. In Europe, the total annual cost of CRC care was estimated at EUR 19.1 billion in 2015 [9]. These costs highlight the importance of implementing cost-effective screening programs globally to detect precancerous lesions early and reduce the economic strain [10]. In Poland, CRC remains one of the most prevalent cancers, ranking as a leading cause of cancer mortality [11].

In 2018, Poland reported approximately 18,000 new CRC cases, with over 10,000 cases among men and about 8000 among women [12]. Mortality rates are substantial, contributing significantly to the nation’s cancer burden [13]. Given the substantial impact of CRC on public health, there is a pressing need for innovative supportive therapies, such as normobaric oxygen (NBO) therapy, to improve patient-reported outcomes and address treatment-related complications.

NBO therapy involves delivering oxygen at atmospheric pressure but at a higher fraction than the ~21% found in ambient air. In contrast, hyperbaric oxygen therapy (HBOT) uses both an elevated oxygen concentration and increased atmospheric pressure (typically ≥1.4 ATA), necessitating specialized pressurized chambers [14]. While HBOT has been studied extensively for conditions such as radiation-induced tissue damage, diabetic foot ulcers, and wound healing, NBO offers a more accessible alternative with fewer risks (e.g., lower incidence of barotrauma, reduced logistical constraints) and may be easier to implement in a variety of clinical settings [15]. Mechanistically, NBO may support erythropoiesis, reduce oxidative stress, and modulate immune function through several pathways. First, by delivering a higher oxygen fraction at normobaric pressure, NBO can alleviate tissue hypoxia—including in the kidneys—which may help preserve or enhance endogenous erythropoietin (EPO) production [16]. Second, the improved oxygenation can potentially reduce the generation of reactive oxygen species (ROS) by optimizing mitochondrial function, thereby attenuating oxidative stress and cellular damage [17]. Third, improved oxygen availability in tissues has been hypothesized to counteract tumor hypoxia, which can otherwise promote immunosuppressive mechanisms (e.g., upregulation of immune checkpoint molecules and recruitment of myeloid-derived suppressor cells) [18,19]. Consequently, NBO could restore or enhance immune cell functionality, potentially improving the anti-tumor immune response in cancer patients [20,21,22].

Recent research has explored the potential benefits of NBO in various medical conditions, including its role in supporting erythropoiesis, reducing oxidative stress, modulating immune function, and improving stress, emotional well-being, and cognitive functions in patients [23].

Anemia is a common complication in cancer patients undergoing chemotherapy, often due to myelosuppression, where the bone marrow’s ability to produce blood cells is suppressed by cytotoxic agents, and reduced EPO production due to kidney damage [16]. EPO is a glycoprotein hormone primarily produced by the kidneys, which stimulates red blood cell formation (erythropoiesis) in the bone marrow. NBO may enhance erythropoiesis under hypoxia conditions by increasing oxygen availability, thereby improving kidney oxygenation and stimulating EPO synthesis [24]. NBO leads to a reduction in oxidative stress in the kidneys that may cause less chemotherapy-induced damage to the kidney parenchyma and thus ensure the viability of EPO-producing cells [25,26]. Hypoxia plays a key role in both normal and pathological cellular processes, influencing EPO gene expression. Extracellular superoxide dismutase (EC-SOD) has been identified as a critical regulator of this response, acting to suppress hypoxia-induced EPO expression. This suggests that superoxide functions as a signaling molecule in the regulation of EPO production, with its downstream effects partially mediated by hypoxia-inducible factor 1-alpha (HIF-1α) [17].

Over the past decade, concerns have grown about the risks of transfusions associated with cancer treatment. Furthermore, no clear evidence has arisen about the benefits of transfusion and questions about increasing mortality have even been raised. The use of a red blood cell progenitor enhancer such as exogenous EPO is extensively recognized, and a relatively low rate of adverse effects has been reported in patients adequately followed in medical institutions. However, the price of such medications is very high and their availability is limited in some countries. A recently described phenomenon called the ‘normobaric oxygen paradox’ (NOP) may show possible clinical applications [27].

Cancer patients often experience significant psychological stress, emotional disturbances, and cognitive impairments due to the disease itself and the side effects of chemotherapy [28]. NBO has shown potential in improving cognitive functions and emotional well-being, which could enhance the overall quality of life for these patients. A preliminary study by Kujawski et al. [29] investigated the effect of NBO on cognitive function in healthy adults. The study demonstrated that NBO sessions led to significant improvements in attention, memory, and executive functions compared with the control group.

These findings suggest that NBO may enhance neural activity and cerebral oxygenation, leading to better cognitive performance. Psychological stress and mood disorders such as depression are common in cancer patients [30]. Bloch et al. [31] conducted a randomized, double-blind, proof-of-concept trial examining the effects of NBO treatment on individuals with mild-to-moderate depression. The study found that NBO significantly reduced depressive symptoms compared with the placebo group. The authors suggested that increased oxygen availability might influence neurotransmitter systems involved in mood regulation, such as serotonin and dopamine pathways.

Reducing stress is a key element supporting cancer treatment, and normobaric therapy can play a significant role. Reduced stress levels improve the function of the immune system. Cortisol can inhibit the activity of some immune cells, so its neutralization, thanks to normobaric therapy, can accelerate the treatment process. The introduction of endorphins and improved mood, as well as general well-being, can significantly increase patients’ motivation to continue mobilizing in treatment. A proper hormonal balance resulting from normobaric therapy contributes to reducing the risk of stress-related complications.

It was revealed that NBO interventions can be effective in supporting mental health. Bloch et al. [32] conducted a pilot study exploring normobaric hyperoxia treatment in patients with schizophrenia. The study administered NBO sessions to patients with schizophrenia and observed improvements in negative symptoms such as blunted affect, social withdrawal, and apathy. Additionally, enhancements in cognitive deficits were noted, particularly in areas like attention, executive functioning, and working memory. These findings indicate that NBO could modulate neural processes underlying schizophrenia, potentially by enhancing cerebral oxygenation and influencing neurotransmitter systems implicated in the disorder. The study suggests that NBO may offer a novel adjunctive treatment modality for schizophrenia, addressing symptoms that are often resistant to conventional therapies [33,34,35].

While HBOT has been extensively studied in oncology, particularly for its benefits in managing radiation-induced tissue injuries and promoting wound healing [36], NBO presents a safer and more accessible alternative, with fewer risks associated with high-pressure exposure [37]. NBO’s potential to improve anemia, reduce oxidative stress, modulate immune function, and enhance cognitive and emotional well-being could synergistically enhance the effectiveness of chemotherapy and improve patient outcomes. Further clinical trials are needed to establish standardized protocols and confirm the efficacy of NBO as a supportive therapy in cancer care. Therefore, this gap in the literature underscores the need for a well-designed, randomized, placebo-controlled trial to systematically evaluate NBO’s impact on anemia, oxidative stress, immune modulation, and psychological well-being in CRC patients undergoing chemotherapy.

The following primary objectives were selected: First, to evaluate the efficacy of NBO in reducing anemia in CRC patients undergoing chemotherapy by measuring markers of erythropoiesis, such as EPO levels, HIF-1α, and complete blood counts. Second, to evaluate the effect of NBO on reducing oxidative stress and improving patients’ cognitive and emotional functions by analyzing levels of cortisol, GABA, dopamine, and other indicators of allostatic load. Third, to analyze the effects of NBO on immune system parameters and reduction in oxidative stress by evaluating a panel of 24 serum cytokines and TNFα levels. Fourth, to identify genetic markers of response to NBO therapy by analyzing the secretome to investigate molecular changes and profiles related to immune function and response to treatment. The effects of NBO on improving patients’ cognitive and emotional functions were quantified simultaneously in the same clinical specimen. In turn, a secondary objective is to assess the impact of NBO on patient-reported outcome measures (PROMs) using standardized tools such as the Perceived Stress Questionnaire (PSQ), Hospital Anxiety and Depression Scale (HADS), and Euro-Quality of Life Questionnaire (EQ-5D).

## 2. Materials and Methods

### 2.1. Study Design and Setting

This project will be a prospective, randomized, double-blind, placebo-controlled clinical trial designed to evaluate the efficacy and safety of NBO therapy in patients diagnosed with CRC undergoing chemotherapy. The study will be conducted in a tertiary hospital setting, ensuring access to standardized treatment protocols and comprehensive patient monitoring. Only NBO operators responsible for therapy administration will be unblinded in order to ensure proper intervention delivery.

### 2.2. Ethical Considerations

This study was approved by the Bioethics Committee at the Lower Silesian Medical Chamber in Wroclaw, Poland (approval no. 06/PN/2025, date: 10 July 2025). The study protocol has been registered at ClinicalTrials.gov (no. NCT06946004, date: 25 April 2025). All patients will be required to give written informed consent before study participation. The study will be conducted in accordance with the guidelines of the Declaration of Helsinki and the principles of Good Clinical Practice (GCP) based on the guidelines of the International Council for Harmonisation of Technical Requirements for Registration of Pharmaceuticals for Human Use (ICH). An up-to-date copy of the study protocol, the current informed consent form for participation in the study, informational documents for study participants, a description of procedures related to participant recruitment, documents related to payments and compensation for study participants resulting from study insurance, and other documents required by the independent Bioethics Committee will be applied. Patients will be informed about voluntary and free participation in the study and about the possibility of withdrawing from the study at any stage without incurring any legal or financial consequences.

### 2.3. Study Participants

The primary study population includes adult patients diagnosed with CRC (ICD10: C18–C20) undergoing chemotherapy. CRC patients commonly experience anemia, immune suppression, and oxidative stress as side effects of chemotherapy, which this study aims to address with NBO therapy. The selected study population is highly relevant due to the significant challenges CRC patients face during chemotherapy, including anemia, oxidative stress, and immune suppression, which impact both treatment outcomes and quality of life. NBO therapy holds potential as a supportive treatment to mitigate these adverse effects.

### 2.4. Eligibility Criteria

Inclusion criteria: (1) age between 18 and 80 years (participants must be adults); (2) diagnosed with CRC (stage II–IV, scheduled for standard chemotherapy (for the study and control groups); (3) baseline hemoglobin levels above 10 g/dL; (4) no concurrent hematologic malignancies; (5) an eastern Cooperative Oncology Group (ECOG) performance status of 0–2 (ensuring that participants are ambulatory and capable of self-care); (6) a life expectancy of at least 12 months (participants are expected to survive for the duration of the study); (7) the ability and a willingness to comply with all study procedures and schedules (including NBO therapy sessions and follow-up visits); (8) adequate organ function as determined by laboratory tests (liver function tests (ALT, AST) and renal function tests (serum creatinine, eGFR)); (9) women of childbearing potential must have had a negative pregnancy test prior to enrollment and agreed to use effective contraception during the study (to ensure safety for potential pregnancies); and (10) written informed consent was obtained prior to any study-related procedures (participants must understand and agree to all aspects of the study).

Exclusion criteria: (1) age under 18 years or over 80 years; (2) severe cardiovascular or respiratory conditions (e.g., unstable angina, recent myocardial infarction, advanced COPD); (3) severe anemia (hemoglobin < 10 g/dL) or any hemolytic disorder that could confound study outcomes; (4) uncontrolled diabetes mellitus (e.g., HbA1c > 8.0% or poor glycemic control requiring frequent hospitalizations); (5) pregnancy or breastfeeding; (6) severe infection or immunocompromised status unrelated to cancer (e.g., advanced HIV infection); (7) current psychiatric or neurological disorders that could interfere with study participation; (8) participation in other investigational therapies within the last 30 days; (9) a lack of written informed consent for study participation; (10) autoimmune or inflammatory conditions (e.g., lupus, rheumatoid arthritis on immunosuppressive therapy) that significantly alter immune responses; and (11) the presence of any contraindication for NBO therapy (e.g., active bleeding, acute infections, inflammation of the optic nerve, epilepsy or seizures, uncontrolled diabetes as noted above, pneumothorax, emphysema, electronic implants).

### 2.5. Randomization and Blinding

Participants will be randomized in a 1:1 ratio to the active NBO (study) or placebo NBO (control) group using a stratified randomization method. Stratification factors include CRC stage (II vs. III vs. IV) and baseline hemoglobin levels (<12 g/dL vs. ≥12 g/dL), as these variables can significantly influence treatment outcomes and the incidence of anemia. A computer-generated allocation sequence will be used within each stratum to ensure an even distribution of these characteristics across the two study arms. Only the NBO operators (who are not involved in data collection or analysis) will have access to the allocation list to administer the assigned intervention. All other study personnel and participants will remain blinded to group assignments.

Patients and clinical staff (excluding NBO operators who administer the therapy) will be blinded to the treatment allocations. Placebo NBO sessions mimic active NBO conditions without therapeutic oxygen adjustments. Both patients and clinical staff involved in patient care and data collection will be blinded to the treatment allocations. NBO operators who administer the therapy will not be blinded in order to ensure the correct delivery of the intervention. They will be trained to maintain confidentiality and avoid any disclosures that could compromise blinding. Both active NBO and placebo NBO sessions will be conducted in chambers that are identical in appearance and setup. This includes similar equipment, procedures, and environmental conditions in order to prevent patients and blinded staff from discerning group assignments.

Unblinding will occur after the database is locked and completion of statistical analysis. Immediate unblinding is permissible in medical emergencies where treatment knowledge is essential for patient care. Unblinding may occur immediately if a serious adverse event or medical emergency arises where knowledge of the treatment allocation is essential for the patient’s safe and effective management. The decision to unblind in an emergency will be made by the principal investigator or a designated medical officer.

### 2.6. Intervention Planned

The intervention will involve NBO as a supportive treatment for CRC patients undergoing chemotherapy. The NBO protocol consists of regular sessions in a normobaric chamber where patients are exposed to controlled oxygen levels aimed at reducing oxidative stress, supporting erythropoiesis, and modulating immune function. Patients in the CRC group will undergo ten NBO sessions across five weeks, with two sessions weekly.

In our study, both the active NBO and placebo NBO groups will undergo sessions in the same normobaric chamber to ensure a consistent experience. For the active NBO group, the chamber will deliver an elevated oxygen concentration (32–40%), while for the placebo group, the oxygen level will be set to approximately 21% (room air). All other environmental parameters—such as chamber pressure, duration of sessions, and ambient conditions—will be identical between the two groups. To maintain the double-blind design, only the NBO operators, who will not be involved in data collection or analysis, will be aware of the specific oxygen settings. Visual indicators of oxygen concentration will be concealed, and the procedure will be standardized so that participants and blinded clinical staff cannot discern which treatment is being administered.

Prior to each NBO session, baseline oxygen saturation (SpO_2_) will be measured using standard pulse oximetry to account for inter-individual variations in oxygenation. If a participant exhibits a significant deviation from their established baseline or displays any clinical signs of respiratory compromise, an arterial blood gas (ABG) analysis will be performed to obtain a more precise measure of oxygenation and guide any necessary adjustments.

#### 2.6.1. Active NBO (Study Group)

Patients will be exposed to NBO conditions in Study Group 1 (aNBO), including oxygen levels of 32–40% (compared with about 21% in the atmosphere), pressure maintained at 1500 hPa (compared with about 1000 hPa outside), carbon dioxide levels between 0.7 and 1.9% (compared with 0.03% in the atmosphere), and hydrogen levels between 0.5 and 1% (which is 10,000 to 20,000 times higher than in the atmosphere). The exposure time in the NBO chamber will be the standard 2 h with an additional 20 min for preparation and adaptation and 10 min for finalization and the decompression period.

#### 2.6.2. Placebo NBO (Control Group)

Patients will be exposed to atmospheric conditions in the same NBO chamber (without normobaric conditions to provide a sham-placebo intervention) in Control Group 2 (pNBO), including oxygen levels at about 21%, pressure maintained at about 1000 hPa, carbon dioxide levels at about 0.03%, and hydrogen levels at about 0.00005% (0.5 parts per million, like in the atmosphere). The primary comparator in this study is the placebo NBO group (n = 127) for CRC patients, which serves to differentiate the specific effects of active NBO from the natural course of treatment without enhanced oxygen exposure.

### 2.7. Research Plan

**Figure 1:**
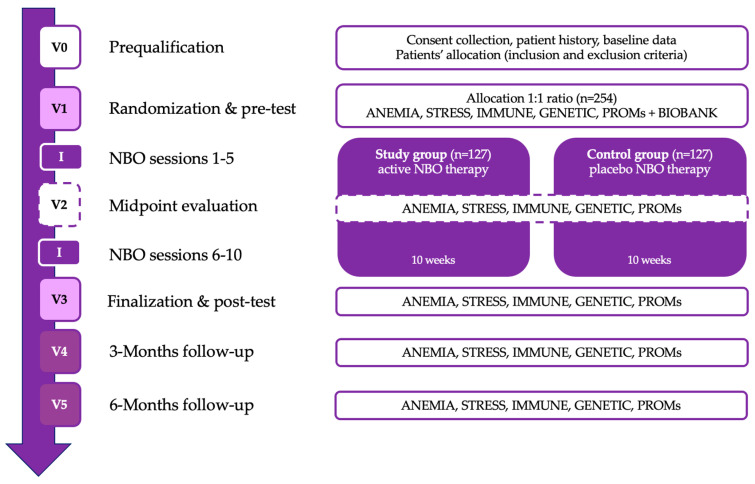
Flow diagram of the study course.

The patient assessment scheme contains six visits from V0 to V5 with subsequent analytical packages with a detailed explanation provided in Section 2.8. The patient assessment schedule consists of six visits, from V0 to V5, each including specific evaluations and interventions. The study will begin with V0 (prequalification/screening), where patient consent will be obtained, medical history will be collected, and baseline data will be recorded. V1 (randomization and pre-test visit) will involve the initial assessments, including anemia, stress, immune function, genetic markers, and patient-reported outcomes (PROMs). Following this, the first phase of the NBO intervention (sessions 1–5) will take place over five weeks. At V2 (midpoint evaluation), the same set of assessments will be repeated to track progress, followed by the second phase of the NBO intervention (sessions 6–10) conducted over the next five weeks. V3 (finalization and post-test visit) will mark the completion of the intervention, with a final round of primary and secondary outcome assessments. The study will then proceed with two follow-up visits: V4 (3 months post-NBO) and V5 (6 months post-NBO), where anemia, stress, immune function, genetic markers, and PROMs will be reassessed to evaluate the long-term effects of the intervention. Table 1 presents the research plan (Figure 1).

### 2.8. Primary Outcomes

#### 2.8.1. ANEMIA Package

This set captures anemia-related biomarkers and systemic effects, including: (1) EPO and HIF-1α (for assessing erythropoiesis and hypoxia response); (2) 5-diff blood morphology (to provide a detailed blood profile); (3) creatinine + eGFR (kidney function markers for a comprehensive evaluation of metabolic status); (3) ALT + AST (liver function markers for a comprehensive evaluation of metabolic status); (5) glucose and insulin (metabolic and glycemic control); (6) lipid panel (overall lipid profile related to systemic health); (7) iron metabolism (TIBC, Iron, Ferritin) and B12 (to evaluate iron stores and deficiency-related anemia); and (8) albumin and CRP (markers of inflammation and nutritional status). In addition to EPO and HIF-1α, we will measure reticulocyte count as a direct indicator of bone marrow response to assess dynamic changes in erythropoiesis.

#### 2.8.2. STRESS Package

This set captures markers related to oxidative stress, hormonal response, and physiological load, including: (1) ROS (measures reactive oxygen species levels); (2) cortisol, GABA, Dopamine, and Serotonin (stress hormones and neurotransmitters); (3) allostatic load (CRP, fibrinogen, HbA1c, DHEA-S, blood pressure (SBP, DBP), and heart rate (HR), to assess chronic stress burden); and (4) anthropometry (BMI, BSA, and WHR, reflecting body composition and risk).

#### 2.8.3. IMMUNO and GENETICS Packages

We will analyze a comprehensive cytokine panel (24-plex or 45-plex) to capture the full spectrum of immune modulation in CRC patients. This panel includes pro-inflammatory (e.g., TNF-α, IL-1β, IL-6), anti-inflammatory (e.g., IL-10), chemotactic (e.g., MCP-1, MIP-1α/β), and growth factors (e.g., VEGF, PDGF) that are collectively relevant for tumor progression, immune cell recruitment, and therapy response in CRC. Multiplex immunoassay technology (e.g., Luminex or similar platforms) will be employed to simultaneously measure these cytokines in a single sample, which enhances throughput, reduces sample volume requirements, and minimizes technical variability compared with individual ELISAs.

### 2.9. Secondary Outcomes

#### 2.9.1. Perceived Stress Questionnaire (PSQ)

The PSQ is dedicated to measuring different dimensions of stress responses. Consisting of 30 items, the PSQ was developed as an instrument for assessing stressful life events and circumstances that tend to trigger or exacerbate disease symptoms. The scale is specifically recommended for clinical settings, though it has been employed in research studies as well. A psychometric evaluation of the scale found internal consistency values ranging from 0.90 to 0.92 and a test–retest reliability of 0.82. In order to complete the PSQ, respondents receive one of two sets of scoring instructions. The general questionnaire queries stressful feelings and experiences over the course of the previous year or two, while the recent questionnaire concerns stress during the last month. Respondents indicate on a scale from 1 (“almost never”) to 4 (“usually”) how frequently they experience certain stress-related feelings. Higher scores indicate greater levels of stress. A total score is found by tallying each item (questions 1, 7, 10, 13, 17, 21, 25, and 29 are positive and are scored according to the directions accompanying the scale). A PSQ index can be found by subtracting 30 from the raw score and dividing the result by 90, yielding a score between 0 and 1 [38].

#### 2.9.2. Hospital Anxiety and Depression Scale (HADS)

The HADS will be used to capture patient perceptions of mental health. It is a widely used screening tool designed to assess anxiety and depression levels in medical patients. Comprising 14 items, it is divided into two subscales: 7 items measure anxiety (HADS-A) and 7 items measure depression (HADS-D). The responses are scored on a 4-point Likert scale, where higher scores indicate greater levels of anxiety or depression. Importantly, the HADS was developed to avoid the confounding effects of physical illness on mood assessments, making it especially suitable for patients in hospital or clinical settings. The total scores for each subscale range from 0 to 21, with thresholds indicating mild, moderate, or severe anxiety or depression. The HADS is extensively used in research and clinical practice, particularly for evaluating psychological well-being in patients with chronic diseases, such as cancer or cardiovascular conditions, as well as during therapeutic interventions like NBO therapy. Its simplicity, reliability, and focus on emotional health make it an effective tool for identifying mental health issues in medically ill patients [39].

#### 2.9.3. EuroQol-5 Dimension (EQ-5D)

The EQ-5D will be used to assess health-related quality of life across five key domains. Each domain reflects a distinct aspect of health, making the EQ-5D a comprehensive measure for tracking changes in overall health and well-being. The EQ-5D contains five domains, each with one question. Mobility measures a patient’s ability to move around. The question may ask, “Are you able to walk about?” Responses range from no problems in walking to being confined to bed. Self-care assesses whether the patient can manage basic personal needs, such as washing and dressing. Responses indicate whether they have no problems, slight problems, moderate problems, severe problems, or are unable to wash or dress. The ‘usual activities’ domain evaluates the patient’s ability to perform everyday activities, including work, study, housework, and leisure activities. Patients indicate if they have no issues, slight problems, moderate issues, severe issues, or extreme difficulties performing these activities. Pain/discomfort assesses the intensity of pain or discomfort the patient is currently experiencing. Responses range from no pain or discomfort to extreme pain or discomfort. Anxiety/depression measures the patient’s level of anxiety or depression, with options from no anxiety/depression to extreme anxiety/depression. Each domain is rated on a scale of 1 to 3 or 1 to 5, depending on the version (EQ-5D-3L or EQ-5D-5L). The scores from each domain are combined into a five-digit number that represents the patient’s health state. Health states can be converted into a single index score using country-specific value sets, which allow researchers to calculate a weighted score for the health state. This index score is often used in economic evaluations of healthcare interventions, like calculating Quality-Adjusted Life Years (QALYs) [40].

### 2.10. Statistical Analysis

Descriptive statistics will be used to summarize study results. Central tendency and dispersion measures will be calculated using parametric or non-parametric methods depending on data distributions. Repeated measures data (V1, V2, V3, V4, and V5) will include ordinal and interval data analyzed with repeated measures ANOVA (parametric) or the Friedman test (non-parametric). Data normality will be verified with the Shapiro–Wilk test. Depending on the distribution, data will be presented as means with standard deviations (parametric) or medians with interquartile ranges (IQRs) (non-parametric). Comparisons between baseline (V1) and follow-up (V4, V5) visits will use the Wilcoxon signed-rank test for paired variables and the Friedman test for multiple comparisons. Post hoc analysis (for significant Friedman tests) will apply Wilcoxon tests with Bonferroni corrections. For between-group comparisons (intervention vs. control), the Mann–Whitney U test will be used.

A comprehensive sample size analysis was conducted to ensure that the study is adequately powered to detect a statistically significant effect of NBO therapy on erythropoiesis stimulation among patients undergoing chemotherapy. The primary endpoint considered was the assumed 40% difference between the active NBO group and the placebo group in the primary outcome measure related to erythropoiesis, such as erythropoietin (EPO) levels. To achieve adequate statistical power, the analysis aimed for 80% power (β = 0.20), indicating a high probability of detecting a true effect if it exists, at a significance level of 5% (α = 0.05), which is the conventional threshold for statistical significance. Effect size estimation was based on Cohen’s (1992) [41] classification, where a small effect size ranges from 0.1 to 0.29, a medium effect size ranges from 0.3 to 0.49, and a large effect size ranges from 0.5 to 1. An effect size corresponding to a medium to large effect was anticipated due to the expected impact of NBO on erythropoiesis.

For the CRC groups (Groups 1 and 2), it was determined that 127 patients per group are required. This accounts for a potential 30% dropout rate, ensuring that the final sample maintains the desired power and accounting for the challenges associated with patient retention over the study period. The sample size calculations were performed using G*Power software (version 3.1.9.7 or the latest version). The parameters used in the calculation included the expected effect size (d), calculated based on the anticipated difference and standard deviation from pilot studies or the existing literature. An allocation ratio (N2/N1) of 1 was used, indicating equal allocation between the active NBO and placebo groups. The test family selected was *t*-tests for means between two independent groups, focusing on the difference between two independent means.

Assumptions made during the calculation included a confidence level of 95% and the use of a two-tailed test, considering the possibility of the effect in both directions. It was also assumed that the variability in outcome measures would be similar across groups, which is important for the validity of the statistical tests used.

The rationale for this sample size is to ensure that the study is sufficiently powered to detect clinically meaningful differences between the active NBO and placebo groups in the primary outcome measures. By accounting for a 30% dropout rate, the study design acknowledges the realities of clinical research involving patients undergoing chemotherapy, who may experience side effects or other factors leading to withdrawal from the study.

An interim analysis will be conducted after 50% of participants have completed the study. The research team will assess data on safety and efficacy trends during this analysis. Based on interim findings, sample size adjustments may be considered, adhering to statistical guidelines to control for Type I error inflation and ensuring the integrity of the study.

Statistical analyses will be performed using software such as SPSS (version 26 or later) and R (version 4.0 or later) with appropriate packages. Supplemental tools like SAS 9.4 M8 or Stata 19 may be used if needed. Data management will involve entering data into a secure database with validation checks to ensure accuracy. Data cleaning procedures will be implemented prior to analysis to address any inconsistencies or errors.

Handling of missing data is an important aspect of the analysis plan. An intention-to-treat (ITT) analysis will be conducted, including all randomized patients in the analysis according to their assigned groups. Missing data will be handled using appropriate statistical methods such as multiple imputation or last observation carried forward (LOCF), depending on the nature and extent of the missing data. Additionally, a per-protocol (PP) analysis will be conducted as a secondary analysis, including only patients who completed the study without major protocol deviations, to assess the robustness of the findings.

Adjustment for multiple comparisons will involve the use of Bonferroni correction or other suitable methods when conducting multiple statistical tests. This approach helps control the overall Type I error rate, reducing the likelihood of false-positive findings due to multiple tests. Sensitivity analyses will also be performed to assess the robustness of the results under different assumptions and statistical models, providing additional confidence in the study conclusions.

### 2.11. Patient Withdrawal

Participants may be withdrawn from the study under specific conditions to ensure safety, data integrity, and adherence to protocol requirements. Voluntary withdrawal allows participants to withdraw consent at any time without penalty or loss of benefits, and they are not required to provide a reason. Medical reasons for withdrawal may arise if the investigator determines that continued participation is not in the patient’s best medical interest due to medical conditions or adverse events that pose a risk. Eligibility non-compliance may lead to withdrawal if post-enrollment evaluation reveals that a participant does not meet inclusion criteria or meets exclusion criteria. Protocol non-adherence, such as a failure to follow study procedures, repeated absences from scheduled visits, or non-compliance affecting data integrity, may result in removal from the study. Pregnancy during the study requires immediate withdrawal for safety reasons. The principal investigator also retains the discretion to withdraw any participant if their continued participation is deemed unsafe or not in their best interest.

### 2.12. Premature Termination

The study may be terminated prematurely under specific circumstances to ensure participant safety, ethical compliance, and data integrity. Safety concerns, such as new or unforeseen adverse events compromising participant safety or an unfavorable risk-to-benefit ratio, may necessitate early termination. Ethical considerations, including violations of ethical standards, Good Clinical Practice (GCP) guidelines, or non-compliance with regulatory requirements, could also lead to study discontinuation. Operational issues, such as insufficient enrollment or significant protocol deviations affecting data validity, may prevent the study from proceeding as planned. In the event of termination, regulatory authorities will be notified within 15 days with a detailed justification, including reports on participant safety and data collected. All participants will be informed promptly, and appropriate follow-up care will be arranged to ensure their well-being.

### 2.13. Safety Evaluation

The safety of participants will be continuously monitored throughout the study to ensure early detection and appropriate management of adverse events (AEs) and serious adverse events (SAEs). Investigators will conduct regular clinical assessments, laboratory evaluations, and vital sign monitoring at each study visit. All AEs and SAEs will be classified according to their severity (mild, moderate, or severe) and their relationship to the intervention (related, possibly related, or unrelated).

The study may be terminated or modified in response to safety concerns, including the emergence of new, unknown adverse reactions that undermine the previously justified benefits of study participation. If new adverse reactions are identified during the study that pose a significant risk to participants, the risk–benefit assessment will be re-evaluated, and continuation of the trial will be reconsidered. Unexpected severe side effects related to NBO therapy or chemotherapy that cannot be managed with standard medical interventions may necessitate protocol modifications or early termination.

In cases where adverse reactions to the therapeutic management reach a level of severity that prevents the trial from continuing safely, the study may be discontinued. Additionally, if the sponsor determines that the study cannot proceed due to a serious violation of Good Clinical Practice (GCP) by an investigator, appropriate actions will be taken, including reporting to regulatory authorities and the ethics committee.

All SAEs will be reported to the ethics committee and regulatory authorities within the required timeframe. An independent Data Safety Monitoring Board (DSMB) will periodically review all safety data to provide recommendations on study continuation, modifications, or termination. Any safety-related findings will be documented in the final study report and considered in future research planning.

## 3. Results

The results of this study will focus on primary outcomes, including changes in erythropoietin (EPO) levels, oxidative stress markers, and immune function parameters, comparing pre- and post-intervention values between the active NBO and placebo NBO groups. Secondary outcomes will assess improvements in patient-reported outcome measures (PROMs), stress reduction, and psychological well-being based on standardized assessment tools, including the Perceived Stress Questionnaire (PSQ), Hospital Anxiety and Depression Scale (HADS), and EuroQol-5D (EQ-5D).

We anticipate that participants in the active NBO group will exhibit greater erythropoiesis stimulation, lower inflammatory cytokine levels, and improved stress adaptation compared with the placebo group. Furthermore, this study may provide insights into the long-term benefits of NBO therapy by analyzing patient outcomes at 3- and 6-months post-intervention.

Patient recruitment in the clinical setting is expected to be completed by 2030. Screening for eligible participants, based on the predefined inclusion and exclusion criteria, will be finalized in 2028. Informed consent will be obtained from all eligible study participants before enrollment, with the first participant scheduled to be enrolled in March 2026. Data analysis is projected to begin in June 2030, followed by the publication of primary and final results.

## 4. Discussion

Chemotherapy-induced anemia, oxidative stress, and immune suppression are significant challenges in the management of CRC, often leading to reduced treatment tolerability and a diminished quality of life. NBO therapy presents a non-invasive and accessible intervention with the potential to synergistically enhance erythropoiesis, reduce oxidative stress, modulate immune responses, and improve psychological well-being, offering a valuable addition to cancer supportive care strategies.

Enhanced oxygen levels can alleviate renal tissue hypoxia, supporting the natural regulation of EPO production. Under normoxic conditions, oxygen-sensitive enzymes promote the hydroxylation and degradation of HIFs, particularly HIF-1α, thereby modulating erythropoiesis. Conversely, in hypoxic states, reduced HIF degradation allows for transcriptional activation of target genes, including EPO, ensuring adequate red blood cell production [42]. Under normoxic conditions, HIF-1α is degraded, but, during hypoxia, it stabilizes and activates the transcription of genes involved in erythropoiesis, including EPO. By modulating oxygen levels through NBO, it is possible to influence HIF-1α activity and subsequently EPO production.

Oxidative stress, resulting from an imbalance between reactive oxygen species (ROS) production and antioxidant defenses, contributes to cellular damage and cancer progression [18,19]. Chemotherapy can exacerbate oxidative stress, leading to increased side effects and decreased quality of life [43]. NBO has been proposed to reduce oxidative stress by enhancing the body’s antioxidant capacity and decreasing ROS levels [44]. It was found that NBO attenuated oxidative-stress-mediated neuronal apoptosis by modulating the Nrf2/ARE signaling pathway [45]. This pathway is crucial for the expression of antioxidant proteins, indicating that NBO could mitigate oxidative damage in cancer patients undergoing chemotherapy.

The immune system is pivotal in controlling cancer progression and determining the effectiveness of therapies [46]. Tumor hypoxia, a condition of low oxygen levels within the tumor microenvironment, fosters immunosuppression by inhibiting the activity of critical immune cells, including T cells and natural killer (NK) cells [18]. This hypoxic state promotes the expression of immune checkpoint molecules, such as programmed death-ligand 1 (PD-L1), on cancer cells, further dampening the anti-tumor immune response [47,48]. Enhancing tissue oxygenation through NBO therapy has been proposed as a strategy to counteract hypoxia-induced immunosuppression. By increasing oxygen availability, NBO may reduce HIF-1α levels, subsequently decreasing PD-L1 expression and restoring immune cell functionality. Studies have demonstrated that supplemental oxygen can alleviate hypoxia-driven immunosuppression, thereby improving the efficacy of immunotherapies in various cancer models. Moreover, hypoxia contributes to the recruitment and activation of immunosuppressive cells, such as regulatory T cells (Tregs) and myeloid-derived suppressor cells (MDSCs), which further inhibit anti-tumor immunity. By normalizing oxygen levels within the tumor microenvironment, NBO therapy may disrupt this immunosuppressive network, enhancing the body’s natural immune response to cancer cells [20,21,22].

The beneficial effects of NBO on cognitive and emotional functions may be attributed to several mechanisms. Enhanced cerebral oxygenation resulting from increased oxygen supply can improve neuronal metabolism and synaptic plasticity, leading to better cognitive performance and neuroprotection [49]. This heightened oxygen availability supports energy-demanding processes in neurons, facilitating learning and memory consolidation. Additionally, NBO may exert neuroprotective effects by reducing oxidative stress in the brain, thereby protecting neurons from damage caused by reactive oxygen species [34]. By mitigating oxidative damage, NBO helps preserve neuronal integrity and function, which is crucial for maintaining cognitive abilities [49]. Furthermore, oxygen therapy might influence the balance of neurotransmitters such as gamma-aminobutyric acid (GABA), glutamate, dopamine, and serotonin, which are essential for mood regulation and cognition. Modulation of these neurotransmitter systems can alleviate symptoms of depression and anxiety, enhancing cognitive functions like attention and executive processing.

Incorporating NBO could offer several advantages for cancer patients experiencing stress, emotional disturbances, and cognitive impairments. By potentially lowering cortisol levels—a primary stress hormone associated with stress responses—and improving mood, NBO may alleviate psychological stress and reduce symptoms of anxiety and depression [31]. This stress reduction can enhance patients’ emotional resilience, helping them cope more effectively with the challenges of cancer treatment. Improved cognitive function resulting from NBO therapy could help patients better manage complex treatment regimens, make informed decisions about their care, and maintain daily activities, thereby promoting independence and quality of life [29]. Addressing emotional and cognitive aspects through NBO not only aids in symptom management but also contributes to holistic cancer care, enhancing patient satisfaction and overall treatment outcomes [50]. By integrating NBO into supportive cancer care, healthcare providers may improve both the psychological well-being and clinical prognosis of their patients.

If the present study found beneficial effects of using NBO in CRC patients, this therapy could emerge as a novel, non-invasive adjunctive treatment to improve chemotherapy outcomes. By enhancing erythropoiesis, reducing oxidative stress, and modulating immune responses, NBO therapy has the potential to alleviate common side effects of chemotherapy, such as anemia and immune suppression. Moreover, its positive impact on psychological well-being and quality of life could empower patients to better cope with the challenges of cancer treatment. These findings may establish a strong foundation for integrating NBO into standard supportive care protocols, ultimately improving patient prognosis and reducing the burden on healthcare systems.

## 5. Conclusions

Chemotherapy-induced anemia, oxidative stress, and immune suppression remain major obstacles in CRC management, often reducing treatment tolerability and quality of life. NBO therapy represents a non-invasive, accessible intervention with the potential to bolster erythropoiesis, mitigate oxidative stress, modulate immune responses, and enhance psychological well-being. By improving oxygen availability, NBO could also help downregulate hypoxia-induced immunosuppression and potentially increase the efficacy of immunotherapy. The resulting improvements in neuronal energy processes and mood regulation further suggest benefits for cognitive and emotional function. If proven effective, NBO could become an important adjunctive therapy in CRC, alleviating treatment-related side effects, improving patient outcomes, and reducing the overall burden on healthcare systems.

## Figures and Tables

**Table 1:** Research plan.

Visit	Phase	Assessments
V0	Prequalification (screening)	Consent, patient history, baseline data
V1	Randomization and pre-test	ANEMIA, STRESS, IMMUNE, GENETIC, PROMs + BIOBANK
NBO sessions 1–5		NBO intervention (first five weeks)
V2	Midpoint evaluation	ANEMIA, STRESS, IMMUNE, GENETIC, PROMs
NBO sessions 6–10		NBO intervention (second five weeks)
V3	Finalization and post-test	ANEMIA, STRESS, IMMUNE, GENETIC, PROMs
V4	3 month follow-up	ANEMIA, STRESS, IMMUNE, GENETIC, PROMs
V5	6 month follow-up	ANEMIA, STRESS, IMMUNE, GENETIC, PROMs

Abbreviations: NBO, normobaric oxygen intervention; V, visit; I, Intervention; ANEMIA, assessment of erythropoiesis markers; STRESS, assessment of stress and oxidative stress; IMMUNO, immunological parameter assessment; GENETIC, analysis of the secretome and molecular changes; PROMs, patient-reported measure outcomes; BIOBANK, collection of human biological material (blood samples) in accordance with the Quality Standards for Polish Biobanks v. 2.00 (2021).

## Data Availability

The data generated in this study will be included in the results of the published article.

## Abbreviations

The following abbreviations are used in this manuscript:

ABG	arterial blood gas
ANEMIA	assessment of erythropoiesis markers
BMI	body mass index
CRC	colorectal cancer
CRP	c-reactive protein
DSMB	Data Safety Monitoring Board
ECOG	Eastern Cooperative Oncology Group Performance Status
eCRF	electronic case report form
EC-SOD	extracellular superoxide dismutase
EPO	erythropoietin
GABA	gamma-aminobutyric acid
GENETIC	analysis of the secretome and molecular changes
GCP	Good Clinical Practice
HADS	Hospital Anxiety and Depression Scale
HbA1c	glycated hemoglobin
HIF-1α	hypoxia-inducible factor 1-alpha
ICH	international council for harmonization
IMMUNO	immunological parameters assessment
NBO	normobaric oxygen
NET	neuroendocrine tumor
NK	natural killer
NOP	‘normobaric oxygen paradox’
MDSCs	myeloid-derived suppressor cells
PD-L1	programmed death-ligand 1
PROMs	Patient-Reported Outcome Measures
PSQ	Perceived Stress Questionnaire
Q-5D	EuroQol-5 Dimension
ROS	reactive oxygen species
SpO_2_	oxygen saturation
STRESS	assessment of stress and oxidative markers
TNFα	tumor necrosis factor-alpha
